# Association between advanced lung cancer inflammation index and cognitive function among US older adults from a cross-sectional survey of the NHANES 2011 to 2014

**DOI:** 10.1097/MD.0000000000044694

**Published:** 2025-09-26

**Authors:** Gaohan Zheng, Lili Zhang, Lin Zhu, Bing Wang, Rui Cao, Xin Liu, Ziyuan Shi, Yuzheng Du

**Affiliations:** a First Teaching Hospital of Tianjin University of Traditional Chinese Medicine, Xiqing District, Tianjin, China; b National Clinical Research Center for Chinese Medicine Acupuncture and Moxibustion, Tianjin, China.

**Keywords:** advanced lung cancer inflammation index, cognitive function, inflammatory response, NHANES, nutritional status

## Abstract

As a composite metric integrating body mass index, serum albumin, and neutrophil-to-lymphocyte ratio, the advanced lung cancer inflammation index (ALI) provides unique advantages for assessing systemic inflammation and nutritional status. This study aimed to systematically investigate the association between chronic inflammation/malnutrition and cognitive decline. Utilizing 2011 to 2014 National Health and Nutrition Examination Survey data, we conducted a cross-sectional analysis. Immediate recall tests (IRT), delayed recall tests (DRT), animal fluency tests, digit symbol substitution tests (DSST), and composite Z-scores were used to evaluate cognitive performance. Stratified interaction analyses, weighted multivariable linear regression, and generalized additive models were employed. Multivariate adjusted models revealed positive correlations between ALI and DSST (β = 1.137, 95% confidence intervals [95% CI]: 1.006–1.285, *P* = .041), DRT (β = 1.201, 95% CI: 1.030–1.400, *P* = .022), and composite scores (β = 1.664, 95% CI: 1.115–2.484, *P* = .016), suggesting that higher ALIs are associated with better cognition. The high-ALI groups performed better in DSST (β = 1.126, 95% CI: 1.028–1.233, *P* = .014), DRT (β = 1.128, 95% CI: 1.003–1.269, *P* = .046), and composite scores (β = 1.528, 95% CI: 1.148–2.034, *P* = .006) than the low-ALI groups. Generalized additive models identified nonlinear dose–response relationships in ALI with immediate recall tests/animal fluency test. Through inflammation/nutrition optimization, ALI may be a modifiable target for preserving cognitive health by enhancing information-processing speed, memory, and composite cognition.

## 1. Introduction

Cognitive function encompasses a wide range of mental abilities, including attention, memory, executive function, and visuospatial ability.^[1]^ Cognitive decline, characterized by a self-perceived decline in cognitive abilities, including memory loss and mental confusion, has become an increasingly significant public health issue in an aging society.^[2]^ In epidemiological studies, mild cognitive impairment (MCI) occurs at a rate of 20 per 1000 person-years among older adults aged 60 to 80,^[3]^ with approximately 35% of MCI patients progressing to dementia or Alzheimer disease (AD) within 5 years.^[4]^ By 2060, the U.S. population aged 65 and older will grow from 52 million to 95 million, increasing their share of the total population from 16% to 23%.^[5]^ The issue of age-related cognitive decline will therefore become even more pressing.^[6]^

Many factors affect the cognitive risk. The latest report identified 14 modifiable cognitive-related risk factors, including smoking, obesity, education, hypertension, and diabetes. Preventing these factors could potentially prevent up to 45% of dementia cases.^[7]^ Nutritional status is closely related to the dietary structure. By affecting energy metabolism, insulin sensitivity, and other ways, a healthy diet can provide the body with nutrients such as omega-3 fatty acids, which can reduce lipid and protein peroxidation.^[8]^ Furthermore, studies indicate that low body mass index (BMI) is a significant risk factor for cognitive impairment in older adults.^[9]^ Among elderly men and African Americans, serum albumin (ALB), a sensitive biomarker of nutritional status, has been associated with improved delayed recall performance.^[10,11]^ Chronic inflammation associated with aging causes persistently elevated levels of pro-inflammatory cytokines,^[12]^ which may cause a neuroinflammatory microenvironment in the central nervous system and contribute to cognitive decline and dementia.^[13]^ Cellular damage is promoted by this pro-oxidative and pro-inflammatory state, which ultimately contributes to cognitive decline.^[14]^

Considering that inflammation and nutrition play complex roles in cognitive decline, a biomarker that can simultaneously assess both factors is needed. Neutrophil-to-lymphocyte ratio (NLR) as an inflammatory marker, serum ALB for nutritional status, and BMI have emerged as highly promising candidates. Advanced lung cancer inflammation index (ALI), calculated from the above common clinical indicators, is proposed as a new clinical index.^[15,16]^ It is currently widely used in the prognostic evaluation of malignancies such as lung cancer and as an indicator of chronic diseases such as hypertension, heart failure, and chronic kidney disease.^[17–20]^ However, research on the association between ALI and cognitive function remains limited. As the first to systematically examine ALI as a composite index, this study offers a theoretical basis for early intervention as it provides a more precise measurement of the interaction between inflammation and nutrition on cognitive health.

## 2. Materials and methods

### 2.1. Study population

**Figure 1.**
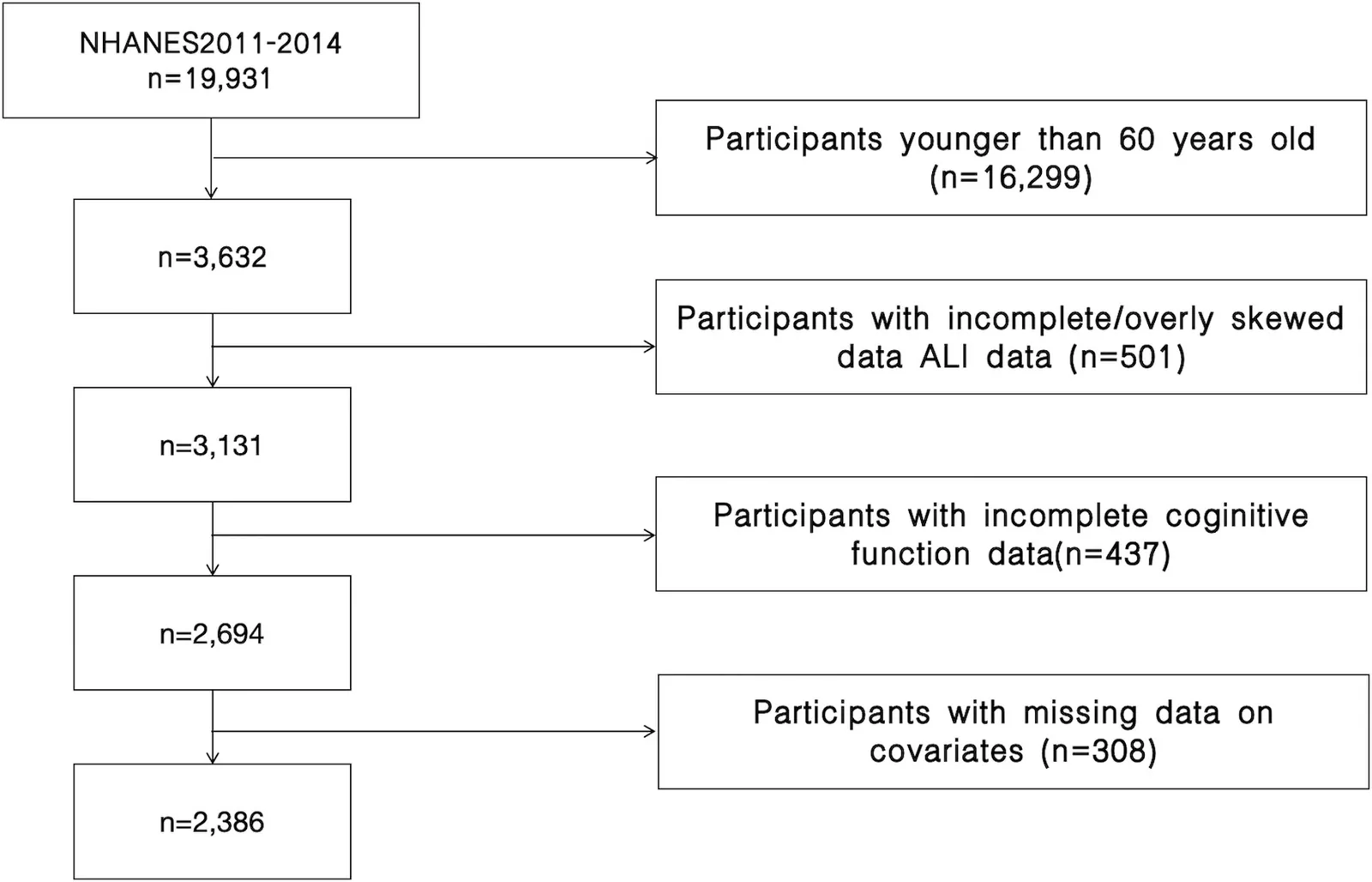
The flow diagram of the sample screening procedure. ALI = advanced lung cancer inflammation index; NHANES = National Health and Nutrition Examination Survey.

This study analyzed data from the to 2011 to 2014 National Health and Nutrition Examination Survey (NHANES), a nationally representative cross-sectional survey conducted by the National Center for Health Statistics using a complex stratified sampling design. The authors did not have access to information that could identify individual participants during or after the data collection. This study strictly adhered to the guidelines outlined in the Strengthening the Reporting of Observational Studies in Epidemiology (STROBE [cross-sectional study checklist]). Since this study did not involve any animal or human experiments, it did not require approval from the Institutional Review Board. In addition, all the data used in this study were derived from public databases. All studies included in the NHANES have obtained ethical approval from the Institutional Review Board, and all participants provided informed consent. From an initial pool of 19,931 participants, we sequentially excluded individuals aged <60 years (n = 16,299), those with missing data required for ALI calculation (BMI, serum ALB, neutrophil or lymphocyte counts, n = 501), those without cognitive assessments (n = 437), and those without complete covariate data (education, marital status, income, smoking, alcohol use, or medical history, n = 308), which resulted in a final analytical sample of 2386 participants (Fig. 1).

### 2.2. Definition and calculation of ALI

ALI has demonstrated significant value in the prognostic assessment of malignancies as a novel inflammation-nutrition composite index. The ALI value was calculated using the following formula: ALI = (BMI × serum ALB)/NLR.^[15]^ We calculated BMI by dividing the weight (kg) by the squared area of the height (m^2^), serum ALB was determined using standard biochemical tests (g/dL), and NLR was calculated by measuring neutrophil to lymphocyte counts(×10^3^cells/μL) by Coulter DxH 800 (Beckman Coulter Inc., Brea) automated hematology analyzer. We excluded extreme outliers exceeding the 99th percentile of ALI values to ensure data quality. For subsequent analyses, ALI values were scaled down by a factor of 100 to enhance the statistical power.

### 2.3. Cognitive function

The immediaterecall tests (IRT), delayed recall tests (DRT), animal fluency test (AFT), and digit symbol substitution test (DSST) were used to evaluate cognitive function in participants aged 60 years and older from 2011 to 2014. In the IRT, adapted from the Consortium to Establish a Registry for AD word learning test, participants were required to learn and recall 10 unrelated words within 3 consecutive trials (score range: 0–30).^[21]^ In the DSST, participants matched symbols to numbers using a reference key within a 2-minute time limit (maximum score: 133).^[22]^ AFT measures categorical verbal fluency and executive function by counting animal names in a minute (score range: 3–39).^[23]^ Using the DRT, administered after AFT and DSST, delayed recall was assessed by reproducing the 10 IRT words (score range: 0–10).^[21]^ To ensure the quality of the tests, all patients consented to the tests and a record of the results was kept. To standardize measurements across scales, raw scores were converted to *Z*-scores (*Z* = (*x* − μ)/σ), with the sum of all 4 *Z*-scores (SUM_*Z*) serving as the composite cognitive function indicator.^[24]^

### 2.4. Covariates

To minimize confounding bias, relevant covariates were identified based on previous studies. NHANES data were collected using standardized questionnaires and physical examinations. The questionnaires covered the following dimensions: demographics such as age and sex, lifestyle factors such as smoking history, drinking history, and history of chronic diseases such as hypertension and diabetes. BMI was calculated by measuring height and weight using anthropometry, which is the main physical examination used in this study. Based on the above-mentioned questionnaires and physical examinations, baseline participant characteristics were calculated, including age, gender, race (Mexican American, non-Hispanic Black, non-Hispanic White, or other); education level (less than high school, high school, or above high school), family status (married, cohabiting, separated, widowed, divorced, or never married), and poverty income based on the poverty income ratio (calculated by dividing family income by the poverty threshold specified by the U.S. Department of Health and Human Services for the corresponding family size), which was classified as ≤1.3 or >1.3. There were 2 types of smoking statuses: never smokers (reported smoking <100 cigarettes in their lifetime) and current smokers (smoked >100 cigarettes in their lifetime). Drinkers and nondrinkers were classified based on the following question: “Have you had at least 12 alcohol drinks/year?” Borderline cases were classified as nondiabetic based on self-reported physician diagnosis. Self-reported physician diagnoses of hypertension were used to define it. Coronary atherosclerotic heart disease (CAD) and cancer status were similarly determined using self-reported physician diagnosis (“Has a doctor ever told you that you have CAD/cancer?”).

### 2.5. Statistical analysis

The Mobile Examination Center weight variable was applied to account for sampling bias in all weighted analyses using the R software (version 4.4.3). Normally distributed continuous variables are presented as mean ± standard deviation, while skewed continuous variables are expressed as medians (interquartile range [P25, P75]). Categorical variables are reported as percentages (%). Multiple linear regression models were developed to examine the relationship between ALI (continuous and categorical variables) and cognitive function outcomes (SUM_*Z*, DSST_*Z*, IRT_*Z*, AFT_*Z*, and DRT_*Z*). The results were expressed as estimated effect sizes (β) and confidence intervals (95% CI). We constructed 3 models: Model 1 (unadjusted), Model 2 (adjusted for age, race, and sex), and Model 3 (all variables adjusted). To assess the potential nonlinear relationships between ALI levels and cognitive test scores, ALI levels were fitted using smooth curves, while other covariates were included linearly. Subgroup analyses were conducted across strata of age, gender, race, education level, marital status, income, smoking status, alcohol consumption, hypertension, diabetes, CAD, and cancer to evaluate potential interactions between ALI and cognitive function. Statistical significance was defined as a 2-sided *P*-value of <.05. Regarding the multiple comparison issue between ALI and the 5 cognitive indicators, we used the Benjamini-Hochberg method to control the false discovery rate (<5%). Statistical significance was defined as a *q*-value of <.05. All analyses accounted for the complex sampling design of NHANES.

## 3. Results

### 3.1. Demographic characteristics

There were 2386 older adults aged ≥ 60 years, with a median age of 68 years, of whom 53.65% were female. As shown in Table 1, the participants were stratified into low (Q1), middle (Q2), and high (Q3) ALI groups based on tertiles. Significant differences in baseline characteristics were observed between the high and low ALI groups in terms of sex, age, ethnicity, marital status, CAD prevalence, ALI, DSST, IRT, DRT, AFT, and composite cognitive score (SUM). In particular, the high ALI group had a younger mean age, higher proportion of females, higher percentage of singles, and lower prevalence of coronary heart disease. In terms of racial distribution, non-Hispanic whites dominated all groups, with the lowest ALI group having the highest proportion (85.69%) of non-Hispanic whites. All 4 standardized cognitive test *Z*-scores and their sum (SUM_*Z*) were significantly higher in the high ALI group than in the low ALI group (*P* < .05), with a 0.93 point increase in the SUM_*Z*.

**Table 1:** Characteristics of all participants according to advanced lung cancer inflammation index groups.

Variable	N	Overall	Q1	Q2	Q3	*P*-value*
Gender	2386					**<.001**
Female		1224 (53.65%)	349 (45.97%)	404 (54.11%)	471 (61.76%)	
Male		1162 (46.35%)	439 (54.03%)	383 (45.89%)	340 (38.24%)	
Age	2386	68.00 (63.00, 74.00)	70.00 (64.00,79.00)	68.00 (64.00,74.00)	66.00 (63.00,71.00)	**<.001**
Race	2386					**<.001**
Mexican		198 (3.08%)	58 (2.64%)	80 (3.63%)	60 (2.97%)	
Black		527 (7.47%)	104 (3.95%)	149 (6.60%)	274 (12.37%)	
White		1209 (81.31%)	485 (85.69%)	401 (82.33%)	323 (75.26%)	
Other		452 (8.14%)	141 (7.72%)	157 (7.44%)	154 (9.39%)	
Education level	2386					.120
Less than high school		550 (14.87%)	173 (15.01%)	181 (14.42%)	196 (15.20%)	
High school		571 (21.90%)	181 (21.97%)	164 (18.66%)	226 (25.43%)	
High school or above		1265 (63.23%)	434 (63.02%)	442 (66.92%)	389 (59.37%)	
Marital status	2386					**.036**
No		995 (34.54%)	334 (34.67%)	295 (30.68%)	366 (38.68%)	
Yes		1391 (65.46%)	454 (65.33%)	492 (69.32%)	445 (61.32%)	
PIR	2386					.600
≤1.3		691 (17.24%)	219 (16.39%)	235 (17.32%)	237 (18.11%)	
>1.3		1695 (82.76%)	569 (83.61%)	552 (82.68%)	574 (81.89%)	
Smoke	2386					.059
No		1172 (49.46%)	355 (44.56%)	383 (50.63%)	434 (53.65%)	
Yes		1214 (50.54%)	433 (55.44%)	404 (49.37%)	377 (46.35%)	
Drink	2386					.140
No		735 (26.73%)	231 (25.20%)	215 (24.57%)	289 (30.83%)	
Yes		1651 (73.27%)	557 (74.80%)	572 (75.43%)	522 (69.17%)	
Hypertension	2386					.600
No		896 (41.44%)	304 (42.84%)	309 (41.66%)	283 (39.61%)	
Yes		1490 (58.56%)	484 (57.16%)	478 (58.34%)	528 (60.39%)	
Diabetes	2386					.800
No		1840 (80.98%)	606 (81.66%)	613 (80.21%)	621 (81.06%)	
Yes		546 (19.02%)	182 (18.34%)	174 (19.79%)	190 (18.94%)	
CAD	2386					**<.001**
No		2170 (90.45%)	684 (85.83%)	727 (92.40%)	759 (93.49%)	
Yes		216 (9.55%)	104 (14.17%)	60 (7.60%)	52 (6.51%)	
Cancer	2386					.140
No		1915 (76.65%)	601 (73.78%)	636 (77.00%)	678 (79.47%)	
Yes		471 (23.35%)	187 (26.22%)	151 (23.00%)	133 (20.53%)	
ALI	2386	55.11 (39.01, 75.70)	33.77 (26.63,39.74)	56.13 (50.47,62.76)	86.77 (77.66, 106.46)	**<.001**
SUM_*Z*	2386	1.10 (−1.27, 3.12)	0.52 (−1.65, 2.43)	1.34 (−1.00, 3.49)	1.45 (−0.85, 3.47)	**<.001**
DSST_*Z*	2386	0.37 (−0.27, 1.01)	0.19 (−0.45, 0.84)	0.49 (−0.22, 1.07)	0.49 (−0.22, 1.13)	**<.001**
IRT_*Z*	2386	0.20 (−0.45, 0.85)	−0.02 (−0.45, 0.63)	0.42 (−0.45, 0.85)	0.42 (−0.45, 0.85)	**.002**
AFT_*Z*	2386	0.21 (−0.52, 0.94)	0.03 (−0.52, 0.76)	0.40 (−0.33, 1.13)	0.21 (−0.52, 0.94)	**.013**
DRT_*Z*	2386	0.43 (−0.44, 0.86)	0.00 (−0.87, 0.86)	0.43 (−0.44, 0.86)	0.43 (−0.44, 0.86)	**<.001**

The bold text signifies a statistically significant distinction with a *P* < .05.

AFT = animal fluency test, ALI = advanced lung cancer inflammation index, CAD = coronary atherosclerotic heart disease, DRT = delayed recall test, DSST = digit symbol substitution test, IRT = immediate recall test, PIR = ratio of family income to poverty.

* For continuous variables, the Kruskal–Wallis rank sum test was used, while for count variables with theoretical numbers <10, Fisher exact probability test was used to obtain the results.

### 3.2. Association between ALI and cognitive function

ALI and cognitive function were analyzed using the weighted multivariable linear regression models presented in Table 2. A fully adjusted model (Model 3) showed a significant positive correlation between the ALI and multiple cognitive function scores. Each unit increase in ALI was associated with a 1.664-point increase in SUM_*Z* (95% CI: 1.115–2.484, *P* = .016), a 1.137-point improvement in DSST_*Z* (95% CI: 1.006–1.285, *P* = .041), and a 1.201-point increase in DRT_*Z* (95% CI: 1.030–1.400, *P* = .022). These associations remained significant when analyzed as categorical variables. The highest ALI tertile group demonstrated significantly higher scores in the DSST_*Z* (β = 1.126, 95% CI: 1.028–1.233, *P* = .014), DRT_*Z* (β = 1.128, 95% CI: 1.003–1.269, *P* = .046), and SUM_*Z* (β = 1.528, 95% CI: 1.148–2.034, *P* = .006) compared to the lowest ALI tertile group.

**Table 2:** Association of the ALI index and different cognitive scores in the multivariate linear regression model.

Cognitive function	Model 1 β (95% CI) *P*-value	Model 2 β (95% CI) *P*-value	Model3 β (95% CI) *P*-value
SUM	2.753 (1.663, 4.557) <.001	1.458 (0.917, 2.318) .107	1.664 (1.115, 2.484) .016
Q1	Ref	Ref	Ref
Q2	2.450 (1.640,3.659) <.001	1.790 (1.269, 2.525) .002	1.714 (1.272, 2.309) .002
Q3	2.482 (1.755, 3.509) <.001	1.422 (1.033, 1.955) .032	1.528 (1.148, 2.034) .006
*P* for trend	< .001	.024	.005
DSST	1.271 (1.086, 1.488) .004	1.092 (0.946, 1.260) .219	1.137 (1.006, 1.285) .041
Q1	Ref	Ref	Ref
Q2	1.251 (1.129, 1.385) <.001	1.148 (1.061, 1.242) .001	1.125 (1.055, 1.199) .001
Q3	1.283 (1.141, 1.442) <.001	1.108 (1.006, 1.221) .039	1.126 (1.028, 1.233) .014
*P* for trend	<.001	.031	.012
IRT	1.359 (1.141, 1.618) <.001	1.080 (0.906, 1.287) .378	1.116 (0.950, 1.311) .168
Q1	Ref	Ref	Ref
Q2	1.270 (1.105, 1.458) .001	1.157 (1.012, 1.322) .034	1.150 (1.012, 1.306) .034
Q3	1.288 (1.141, 1.454) <.001	1.079 (0.948, 1.228) .236	1.097 (0.965, 1.247) .144
*P* for trend	< .001	.214	.130
AFT	1.100 (0.914, 1.324) <.301	1.056 (0.878, 1.271) .547	1.092 (0.900, 1.324) .348
Q1	Ref	Ref	Ref
Q2	1.245 (1.078, 1.437) .004	1.189 (1.046, 1.353) .01	1.171 (1.036, 1.324) .015
Q3	1.144 (0.995, 1.315) .059	1.073 (0.934, 1.234) .306	1.097 (0.957, 1.257) .170
*P* for trend	.046	.260	.143
DRT	1.448 (1.228, 1.708) <.001	1.171 (1.002, 1.368) .048	1.201 (1.030, 1.400) .022
Q1	Ref	Ref	Ref
Q2	1.239 (1.070, 1.435) .006	1.134 (0.982, 1.310) .085	1.132 (0.977, 1.311) .093
Q3	1.314 (1.160, 1.487) <.001	1.108 (0.983, 1.248) .089	1.128 (1.003, 1.269) .046
*P* for trend	< .001	.081	.041

Model 1: no adjustment.

Model 2: adjusted for age, gender, ethnicity.

Model 3: adjusted for age, gender, ethnicity, educational level, marital status, ratio of family income to poverty, smoke, drink, hypertension, diabetes, coronary atherosclerotic heart disease and cancer.

AFT = animal fluency test, DRT = delayed recall test, DSST = digit symbol substitution test, IRT = immediate recall test.

**Figure 2.**
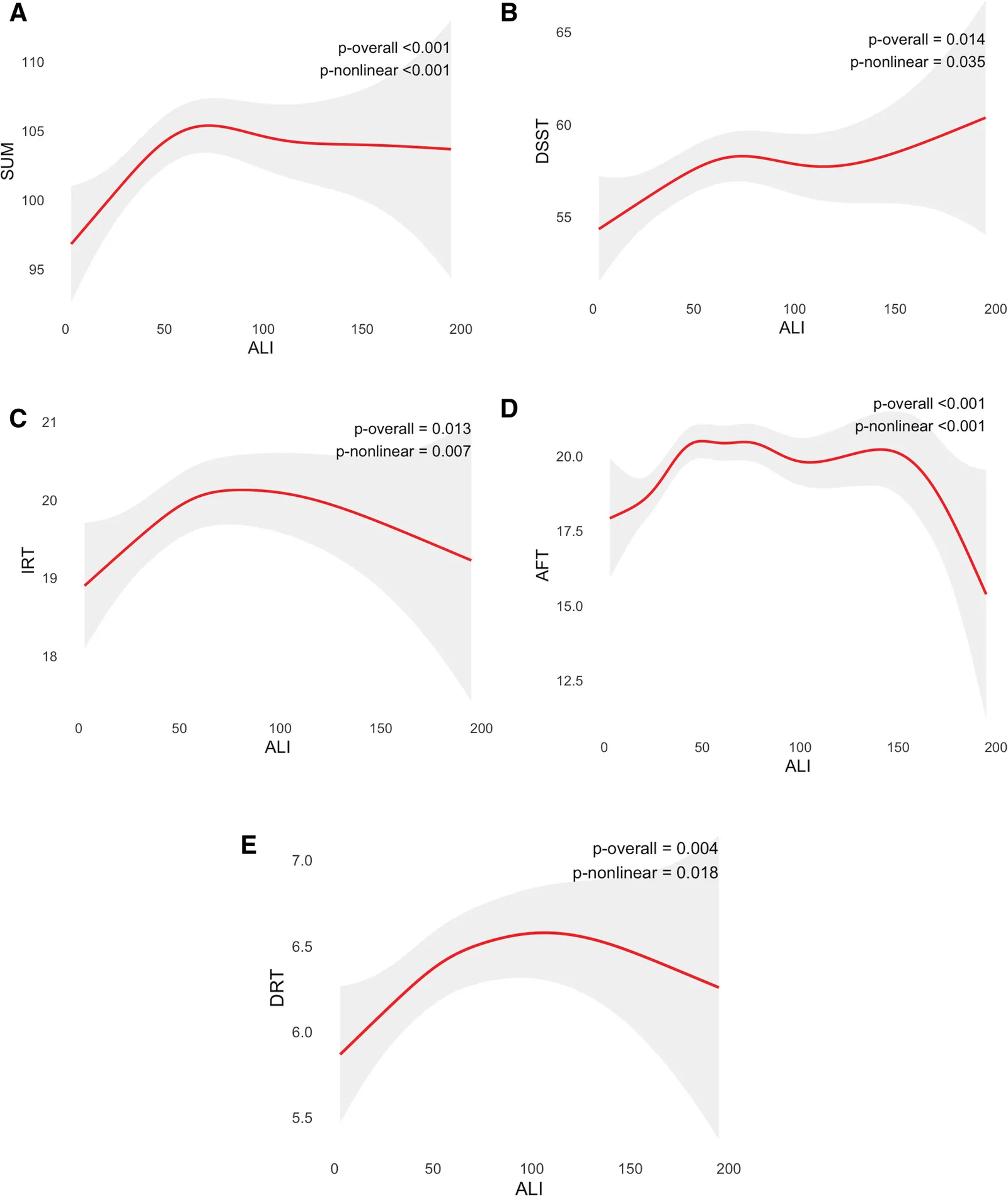
Generalized additive model of the association between ALI and different cognitive scores. (A) Generalized additive model of the association between ALI and SUM; (B) generalized additive model of the association between ALI and DSST; (C) generalized additive model of the association between ALI and IRT; (D) generalized additive model of the association between ALI and AFT; (E) generalized additive model of the association between ALI and DRT. AFT = animal fluency test, ALI = advanced lung cancer inflammation index, DRT = delayed recall test, DSST = digit symbol substitution test, IRT = immediate recall test. The solid red lines represent the smooth curve fit between the variables, whereas the gray areas indicate the 95% confidence interval derived from this fit.

However, neither IRT_*Z* nor AFT_*Z* showed a statistically significant linear association with ALI. To further explore the potential complex relationships, we constructed generalized additive models (Fig. 2), which revealed significant nonlinear associations between ALI and various cognitive domains. Specifically, ALI demonstrated an S-shaped curve relationship with DSST (inflection points: 72.66 and 113.4, Fig. 2B); inverted U-shaped relationships with SUM, IRT, and DRT (inflection points: 70.72, 80.42, and 105.64, respectively, in Fig. 2A, C, and E); and an M-shaped relationship with AFT (inflection points: 49.38, 103.7, and 140.55 in Fig. 2D).

### 3.3. Subgroup analysis and interaction effects

**Figure 3.**
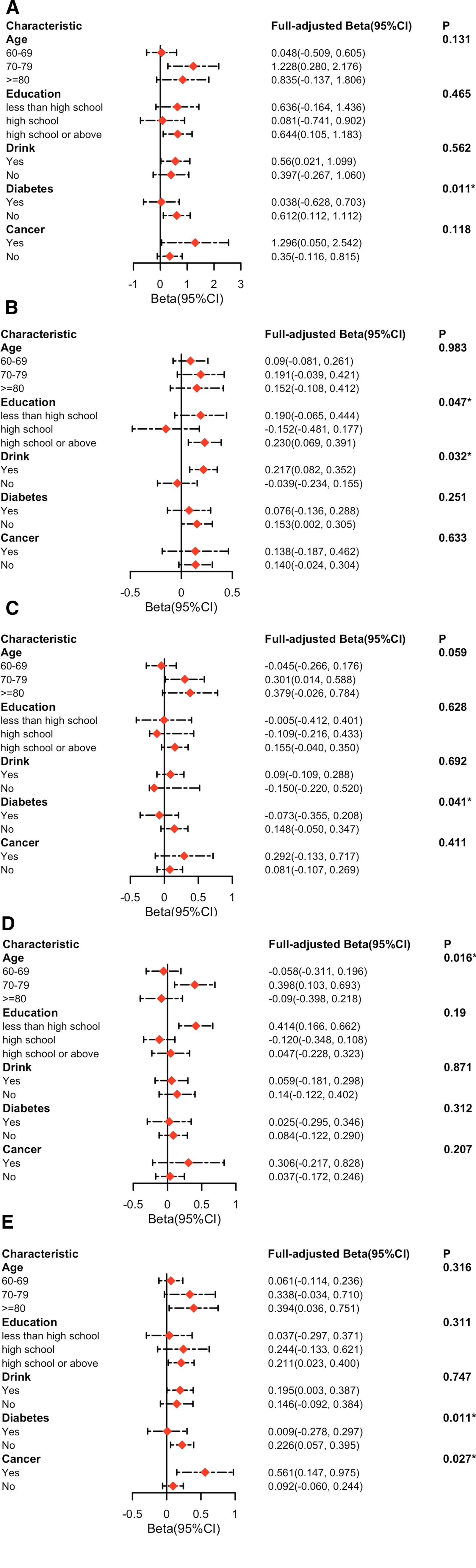
Subgroup analysis of the association between ALI and different cognitive scores. (A) Subgroup analysis of the association between ALI and SUM; (B) subgroup analysis of the association between ALI and DSST; (C) subgroup analysis of the association between ALI and IRT; (D) subgroup analysis of the association between ALI and AFT; (E) subgroup analysis of the association between ALI and DRT. AFT = animal fluency test, ALI = advanced lung cancer inflammation index, DRT = delayed recall test, DSST = digit symbol substitution test, IRT = immediate recall test. Age, education level, alcohol consumption, diabetes, and cancer variables were significant in some subgroup analyses.

As shown in Fig. 3, stratified analyses across different population characteristics revealed significant modification effects. Nondiabetic participants exhibited stronger positive correlations between ALI and both SUM_*Z (P* = .011 in Fig. 3A) and DRT (*P* = .011 in Fig. 3E), whereas those with higher education (*P* = .047) and alcohol consumption (*P* = .032) demonstrated stronger associations between ALI and DSST (Fig. 3B). An age-stratified analysis revealed a significantly stronger correlation between ALI and AFT in individuals 70 to 80 years old (*P* = .016, Fig. 3D) and a distinct relationships between ALI and DRT in cancer patients (*P* = .027, Fig. 3E). In addition, the association between ALI and cognitive function was not significant in the other subgroups (*P* > .05).

## 4. Discussion

For the first time, a systematic evaluation of the association between ALI and different cognitive function scores was conducted with 2386 participants. After adjusting for potential confounders affecting cognition, our cross-sectional analysis revealed a significant positive correlation between ALI and cognitive performance, especially in the tests of information processing and memory (DSST and DRT) and composite cognitive function. ALI selectively influences distinct cognitive domains, and its effects are likely to change dynamically depending on the level of ALI. In contrast, no statistically significant linear association was observed between ALI and IRT or AFT scores. However, smoothed curve fitting revealed nonlinear relationships, perhaps due to the varying sensitivity thresholds of cognitive tests or to the heterogeneity of baseline health status and lifestyle factors.

The relationship between body weight and cognitive function exhibits a unique “obesity protective effect” in the elderly. According to Ren et al, moderate overweight status is associated with reduced cognitive impairment.^[25]^ According to the latest AD prevention guidelines, older adults aged 65 and older should avoid being underweight.^[26]^ A large-scale Chinese study (n = 12,027) found that overweight status, as defined by BMI, reduced cognitive impairment risk among the elderly by 16%.^[27]^ In addition to their reduced muscle mass, underweight older adults are more likely to develop underlying diseases or nutritional deficiencies, which may contribute to this protective effect.^[28]^ Secondly, improving glucose metabolism may reduce cognitive impairment risk in the elderly through increased leg fat mass.^[29]^ In addition, higher BMI can improve cognitive function in the elderly by elevating insulin-like growth factor-1, leptin, and estrogen levels.^[30–32]^ Lastly, serum urate, which acts as an antioxidant, is positively correlated with BMI and slows the progression of neurodegenerative diseases.^[33]^

These results indicate a positive effect of obesity on cognitive function. However, some studies have reported the negative effects of obesity on cognitive function. In a study of 28,867 U.S. participants, obese individuals had significantly lower composite cognitive scores than normal-weight controls.^[34]^ In line with our findings, domain-specific analyses identified executive function, working memory, and verbal learning as the most vulnerable cognitive domains in obesity.^[35,36]^ Additionally, neuroimaging studies revealed striking overlaps between obesity-related brain changes and AD-vulnerable regions, especially cortical thinning in the temporoparietal and frontal lobes.^[37]^ Neurobiological evidence supports obesity as an independent dementia risk factor based on these parallels in cognitive and neuroanatomical profiles.^[38]^ This difference in results can explain our conclusion. According to smoothed curve fitting, cognitive performance declines when ALI exceeds a certain level. Researchers have found a positive correlation between carbohydrate intake and cognitive impairment/dementia.^[39]^ Hyperglycemia-induced advanced glycation end products and elevated oxidative stress may contribute to cognitive deficits and amyloid formation.^[40]^ Li et al also reported that excessive copper intake reduced cognition by increasing ceruloplasmin levels and oxidative stress.^[41]^ Thus, excessive nutritional intake may paradoxically increase oxidative stress and adversely affect cognition.

Early studies have indicated that serum ALB is associated with nutritional status.^[10]^ There is also a positive correlation between serum ALB levels and cognitive function in elderly populations, with hypoalbuminemia potentially accelerating cognitive decline.^[11,42]^ Thus, lower serum ALB levels may reflect malnutrition and could be linked to cognitive decline. Hypoalbuminemia may adversely affect cognitive function because it impairs the ability to repair and maintain brain tissue, ultimately reflecting nutritional deficiencies throughout the body.^[43]^ Misfolded serum ALB, commonly found in elderly patients with comorbid conditions, may also exacerbate cognitive decline.^[44]^ ALB plays pivotal roles in antioxidant defense, anti-inflammatory processes, and signal transduction in bodily fluids.^[45]^ Because of its unique molecular structure (cysteine residue at position 34), ALB is an ideal biomarker for assessing systemic oxidative stress.^[46]^ ALB mitigates excessive oxidative stress triggered by inflammatory responses in aging neurons by acting as a primary plasma transport protein and an extracellular antioxidant. As a result of these properties, it is an excellent biological marker for assessing cognitive impairment in the elderly.^[47]^ Notably, ALB critically maintains plasma colloid osmotic pressure and circulatory blood volume.^[48]^ Cognitive impairment may result from its deficiency, as it may compromise cerebral perfusion. Additionally, experimental studies show that ALB binds β-amyloid protein specifically, which reduces neurotoxicity and inhibits protein aggregation, conferring neuroprotection.^[49]^ Through a variety of physiological pathways, ALB plays a multifaceted role in cognitive regulation.

In recent years, the role of chronic inflammation in cognitive decline has garnered increased attention. In animal studies, it has been demonstrated that elderly mice are more likely to be affected by systemic inflammatory responses, with cognitive dysfunction models displaying abnormalities in oxidative stress and cytokine secretion,^[50]^ which provides direct evidence linking neuroinflammation to age-related cognitive decline. In addition, studies have shown that neuroinflammation is precisely caused by glial cell activation and inflammatory factors produced by neurons affected by senile plaques in elderly AD patients with.^[51]^ Using animal models and gene expression profiles, Sobue et al concluded that prolonged neuroinflammation impairs microglial homeostatic function, which in turn contributes to neuronal degeneration.^[52]^ The homeostasis of the immune system is crucial for maintaining normal neurocognitive function.^[53]^ It is noteworthy that NLR has been closely associated with clinical outcomes in various cardiovascular and cerebrovascular diseases because it reflects the balance between innate (neutrophils) and adaptive (lymphocytes) immunity.^[54,55]^ Peripheral blood NLR levels are significantly higher in AD patients than in those with MCI,^[56]^ consistent with findings by Guo et al that show inverse correlations between systemic inflammatory markers and cognitive performance.^[57]^ Our study’s conclusions are further supported by these observations. Multiple mechanisms might explain the detrimental effects of systemic inflammation on cognition. First, although microglial activation aids in clearing amyloid plaques, a neuroprotective response against neurodegeneration-chronic inflammation downregulates β-amyloid protein receptors and degrading enzymes, impairing microglial phagocytosis.^[58,59]^ Furthermore, persistent inflammation reduces cerebral blood flow and oxygenation, ultimately impairing neuronal activity and cognitive function.^[60]^ Studies have also shown that disruption of the blood–brain barrier by inflammatory factors or the release of neurotoxic substances can directly affect the brain, precipitating neuroinflammation.^[61]^

ALI offers distinct advantages over complex neuropsychological assessments and neuroimaging from a clinical perspective. ALI serves as an early warning system for cognitive decline and is a blood–based biomarker that can be easily measured. The clinical goal of delaying cognitive decline is to facilitate targeted cognitive monitoring and timely intervention.

This study has several strengths. As a first step, we demonstrated the relationship between cognitive function and ALI in adults aged 60 years or older, providing a novel and easily accessible biomarker for investigating nutritional/inflammatory status and cognitive function. In addition, the use of nationally representative epidemiological survey data minimized heterogeneity, while the comprehensive assessment of cognition (DSST, IRT, AFT, and DRT) and composite score (SUM_Z) minimized heterogeneity. Multiple statistical approaches (weighted multivariate regression, generalized additive model) have enabled the systematic evaluation of the dose–response relationship of ALI across cognitive domains. However, some limitations of this study warrant consideration. A prospective cohort study is needed to validate the causal relationship between ALI and cognitive function owing to its cross-sectional design. The findings may not be generalizable to other ethnicities or age groups due to the exclusive elderly population in the U.S. Although major confounders were adjusted, residual confounding may have persisted. Further studies are required to confirm ALI’s diagnostic advantages of ALI over conventional inflammatory and nutritional markers.

## 5. Conclusions

ALI was significantly associated with specific cognitive domains in the elderly population, including information processing speed, delayed memory function, and composite cognitive function. Cognitive function in these domains positively correlated with elevated ALI levels.

## Acknowledgments

We express our gratitude to the staff at the National Center for Health Statistics for their work in designing, collecting, managing, and publicly sharing the NHANES data.

## Author contributions

**Conceptualization:** Gaohan Zheng.

**Formal analysis:** Lili Zhang, Lin Zhu, Bing Wang, Rui Cao, Xin Liu, Ziyuan Shi.

**Funding acquisition:** Yuzheng Du.

**Methodology:** Lili Zhang.

**Resources:** Yuzheng Du.

**Supervision:** Yuzheng Du.

**Writing – original draft:** Gaohan Zheng.

**Writing – review & editing:** Lili Zhang, Lin Zhu, Bing Wang, Rui Cao, Xin Liu, Ziyuan Shi.
